# Supplementary material for: Ecological, Social, and Other Environmental Determinants of Dengue Vector Abundance in Urban and Rural Areas of Northeastern Thailand
Source: Int J Environ Res Public Health. 2021 Jun 2;18(11):5971. doi: 10.3390/ijerph18115971 (PMC8199701; doi:10.3390/ijerph18115971)
Supplement: Supplementary file 1 [file ijerph-18-05971-s001.zip › ijerph-1213494-supplementary.pdf]

Additional file 1: Table S1. Summarized knowledge characteristics regarding climate change among study populations in selected urban and rural villages in northeastern Thailand (percentages in parentheses)

Factors	**Urban**	**Rural**	**Total**	x^2 (P $value$)
N (No of households)	**64**	**64**	**128**	
**<u>Beliefs, understanding & awareness about climate change and its connection to dengue?</u>**				
**K1. Have you heard about climate change?**				2.9 (.08)
No	2 (3.1)	7 (10.9)	9 (7.0)	
Yes	61 (95.0)	58 (90.0)	119 (93.0)	
**K2. Do you believe the climate is changing?**				.09 (.75)
No	5 (7.8)	6 (9.4)	11 (8.6)	
Yes	58 (91.2)	57 (89.0)	117 (91.4)	
**K3. Understanding and awareness about climate change**				.05 (.97)
Good	24 (37.5)	23 (35.9)	47 (36.7)	
Medium	27 (42.2)	27 (42.2)	54 (42.2)	
Poor	13 (20.3)	14 (21.9)	27 (21.1)	
**K4. Do you think changes in climate can affect dengue fever and its vector?**				1.9 (.16)
**No**	14 (21.9)	21 (32.8)	35 (27.3)	
**Yes**	50 (78.1)	43 (67.2)	93 (72.7)	
**<u>Local & global climate change problem</u>**				
**K5. What type of change to the climate do you think has happened in your area?**				
Excessive temperature	63 (98.4)	61 (95.3)	124 (96.9)	1.03 (.31)
Change of pattern of rainfall	36 (56.3)	33 (51.6)	69 (53.9)	.28 (.59)
**K6. What have you already heard about the possible future effects of climate change and topic of global climate change and changing mosquito habitat suitability)?**				
More rain	17 (26.6)	19 (29.7)	36 (28.1)	.15 (.69)
Hotter	54 (84.4)	48 (75.0)	102 (79.7)	1.7 (.18)
More floods	17 (26.6)	16 (25.0)	33 (25.8)	.04 (.84)
**K7. What do you think is the biggest local climate change problem?**				
Increase in extreme weather (more heat/ very cold/ heavy or torrential rainfall)	46 (71.9)	41 (64.1)	87 (68.0)	.89 (.34)
Floods	9 (14.1)	6 (9.4)	15 (11.7)	.68 (.41)
**Summarized knowledge level (good vs poor)**				1.06 (.30)
Poor	46 (71.9)	51 (79.7)	97 (75.8)	
Good	18 (28.1)	13 (20.3)	31 (24.2)	

All P-values are based on a Chi-square test or Fisher's exact test (as appropriate) for independence analysis based on knowledge characteristics regarding climate change (in respective study sites, separately). Knowledge scores considered (≥80 = good and <80 = poor).

Additional file 2: Table S2. Summarized attitude characteristics regarding climate change among study populations in selected urban and rural villages in northeastern Thailand (percentages in parentheses)

Factors	Urban	Rural	Total	x^2 (P $value$)
N (No of households)	**64**	**64**	**128**	
**A1. How serious a problem do you think climate change and more spread of dengue fever at this moment?**				1.02 (.60)
Not serious	10 (15.6)	12 (18.8)	22 (17.2)	
Moderately serious	20 (31.3)	15 (23.4)	35 (27.3)	
Extremely serious	34 (53.1)	37 (57.8)	71 (55.5)	
**A2. Are you concerned that Climate Change is causing more dengue fever in your area?**				.77 (.38)
No	11 (17.2)	15 (23.4)	26 (20.3)	
Yes	53 (82.8)	49 (76.6)	102 (79.7)	
**A3. Do you think we can reduce dengue risk due to climate change?**				3.51 (.06)
We can and must	57 (89.1)	49 (76.6)	106 (82.8)	
We can't change	7 (10.9)	15 (23.4)	22 (17.2)	
**A4. Should media take a leading role in raising awareness about climate change and dengue risk reduction and prevention issues?**				.06 (.79)
No	8 (12.5)	9 (14.1)	17 (13.3)	
Yes	56 (87.5)	55 (85.9)	111 (86.7)	
**A5. Do you think the government should do something to mitigate dengue risk that may spread due to climate change?**				13.3 (.000)
**No**	14 (21.9)	34 (53.1)	48 (37.5)	
Yes	50 (78.1)	30 (46.9)	80 (62.5)	
**A7. Need for better awareness and knowledge on climate change?**				3.9 (.04)
No	2 (3.1)	8 (12.5)	10 (7.8)	
Yes	62 (96.9)	56 (87.5)	118 (92.2)	
**A8. Would you like to personally receive information about climate change updates and how to protect yourself from dengue fever?**				1.8 (.17)
No	4 (6.3)	1 (1.6)	5 (3.9)	
Yes	60 (93.8)	63 (98.4)	123 (96.1)	
**Summarized attitude level (good vs poor)**				1.62 (.20)
Poor	21 (32.8)	28 (43.8)	49 (38.3)	
Good	43 (67.2)	36 (56.3)	79 (61.7)	

All *P*-values are based on a Chi-square test or Fisher's exact test (as appropriate) for independence analysis based on attitude characteristics regarding climate change (in respective study sites, separately). Attitude scores considered (≥80 = good and <80 = poor).

Additional file 3: Table S3. Summarized practice characteristics regarding climate change among study populations in selected urban and rural villages in northeastern Thailand (percentages in parentheses)

Factors	Urban	Rural	Total	x^2 $(P\ value)$
N (No of households)	**64**	**64**	**128**	
**P1. Have you taken any actions to prepare and reduce dengue risk due to climate change?**				11.03 (.004)
Yes, many	18 (28.1)	29 (45.3)	47 (36.7)	
Some/few	45 (70.3)	28 (43.8)	73 (57.0)	
No, nothing	1 (1.6)	7 (10.9)	8 (6.3)	
**P2. During the last year, have you done anything about changes in mosquito control practice following an extreme event and prevent dengue risk?**				.43 (.51)
No	6 (9.4)	4 (6.3)	10 (7.8)	
Yes	58 (90.6)	60 (93.8)	118 (92.2)	
**P3. Kinds of practice following an extreme event and prevent dengue risk?**				
Cleaned drains	38 (59.4)	35 (54.7)	73 (57.0)	.28 (.59)
Cleaned or helped to maintain public drainage systems from waste	44 (68.8)	35 (54.7)	79 (61.7)	2.6 (.10)
**P4. Do you or your household members use the internet?**				3.08 (.07)
No	9 (14.1)	17 (26.6)	26 (20.3)	
Yes	55 (85.9)	47 (73.4)	102 (79.7)	
**P5. Which of the following modes of information collection and communication tools you use for climate literacy (adaptation and mitigation capacities)?**				
Newspaper	8 (12.5)	3 (4.7)	11 (8.6)	2.48 (.11)
TV (local/International)	63 (98.4)	52 (81.3)	115 (89.8)	10.3 (.01)
Radio	14 (21.9)	6 (9.4)	20 (15.6)	3.79 (.01)
Social Media (Facebook, Line, Instagram, etc.)	42 (65.6)	34 (53.1)	76 (59.4)	2.07 (.15)
**P6. How often you use the above modes of communication**				7.2 (.02)
Everyday	62 (96.9)	54 (84.4)	116 (90.6)	
Some times	2 (3.1)	4 (6.3)	6 (4.7)	
Never use	0 (0.0)	6 (9.4)	6 (4.7)	
**Summarized practice level (good vs poor)**				2.59 (.10)
Poor	49 (75.6)	56 (87.5)	105 (82.0)	
Good	15 (23.4)	8 (12.5)	23 (18.0)	

All *P*-values are based on a Chi-square test or Fisher's exact test (as appropriate) for independence analysis based on practice characteristics regarding climate change (in respective study sites, separately). Practice scores considered (≥80 = good and <80 = poor).

Additional file 4: Table S4. Summarized knowledge characteristics regarding dengue among study populations in selected urban and rural villages in northeastern Thailand (percentages in parentheses)

Factors	Urban	Rural	Total	x^2 (P $value$)
N (No of households)	**64**	**64**	**128**	
**Knowledge, Understanding and awareness about dengue**				
**Transmission of dengue**				
K1. Dengue is caused by a virus	49 (76.6)	50 (78.1)	99 (77.3)	.04 (.83)
K2. Dengue virus has four serotypes	23 (35.9)	30 (46.9)	53 (41.4)	1.5 (.20)
K3. A person is vulnerable/can get dengue more than once	51 (79.7)	47 (73.4)	98 (76.6)	.69 (.40)
K4. Ae. aegypti and *Ae. albopictus* are the vectors of dengue	42 (65.6)	21 (32.8)	63 (49.2)	13.78 (.000)
K5. Do *Aedes* mosquitoes transmit DF	53 (82.8)	59 (92.2)	112 (87.5)	2.5 (.10)
**symptoms and signs of dengue**				
K6. Fever	58 (90.6)	57 (89.1)	115 (89.8)	.08 (.77)
K7. Headache	39 (60.9)	41 (64.1)	80 (62.5)	.13 (.71)
K8. Joint pains	33 (51.6)	32 (50.0)	65 (50.8)	.03 (.86)
K9. Muscle pain	33 (51.6)	34 (53.1)	67 (52.3)	.03 (.86)
K10. Pain behind the eyes	31 (48.4)	30 (46.9)	61 (47.7)	.03 (.86)
K11. Nausea/vomiting	34 (53.1)	26 (40.6)	60 (46.9)	2.0 (.15)
**K12. Most frequent bite time of mosquitoes (Day time)**				1.03 (.30)
No	7 (10.9)	11 (17.2)	18 (14.1)	
Yes	57 (89.1)	53 (82.8)	110 (85.9)	
**Vector morphology (identification)**				
K13. Can you identify *Aedes* mosquitoes?	40 (62.5)	36 (56.3)	76 (59.4)	.51 (.47)
Have white spots on their legs	34 (53.1)	15 (23.4)	49 (38.3)	11.93 (.001)
**Vector breeding (places)**				
K14. Breed in standing water	60 (93.8)	57 (89.1)	117 (91.4)	.89 (.34)
K15. Breed in clean water	47 (73.4)	53 (82.8)	100 (78.1)	1.64 (.20)
K16. Breed in leaf axils and plant surfaces	33 (51.6)	36 (56.3)	69 (53.9)	.28 (.59)
K17. Breed in water retention tanks in K18. A\C machines and refrigerators	40 (62.5)	34 (53.1)	74 (57.8)	1.15 (.28)
K19. In the abandoned tyres	62 (96.9)	63 (98.4)	125 (97.7)	.34 (.55)
**Summarized knowledge level** (good vs poor)				.28 (.59)
Poor	35 (54.7)	38 (59.4)	73 (57.0)	
Good	29 (45.3)	26 (40.6)	55 (43.0)	

All *P*-values are based on a Chi-square test or Fisher's exact test (as appropriate) for independence analysis based on knowledge characteristics regarding dengue (in respective study sites, separately). Knowledge scores considered (≥80 = good and <80 = poor).

Additional file 5: Table S5. Summarized attitude characteristics regarding dengue among study populations in selected urban and rural villages in northeastern Thailand (percentages in parentheses)

Factors	**Urban**	**Rural**	**Total**	x^2 (P $value$)
N (No of households)	**64**	**64**	**128**	
**A1. Controlling of the breeding places of mosquitoes a good strategy to prevent DF?**				.05 (.82)
No	13 (20.3)	12 (18.8)	25 (19.5)	
Yes	51 (79.7)	52 (81.3)	103 (80.5)	
**A2. Communities should actively participate in controlling the vectors of DENV?**				1.44 (.23)
No	20 (31.3)	14 (21.9)	34 (26.6)	
Yes	44 (68.8)	50 (78.1)	94 (73.4)	
**A3. Change the water in plant pot trays every week**				5.8 (.05)
Extremely confident	54 (84.4)	47 (73.4)	101 (78.9)	
Moderately confident	8 (12.5)	7 (10.9)	15 (11.7)	
Not at all	2 (3.1)	10 (15.6)	12 (9.4)	
**A4. Clean the drain from blockage every 7 days**				10.3 (.006)
Extremely confident	52 (81.3)	41 (64.1)	93 (72.7)	
Moderately confident	10 (15.6)	9 (14.1)	19 (14.8)	
Not at all	2 (3.1)	14 (21.9)	16 (12.5)	
**A5. I can always cover tightly all water containers inside and outside house**				2.17 (0.33)
Extremely confident	49 (76.6)	45 (70.3)	94 (73.4)	
Moderately confident	13(20.3)	13 (20.3)	26 (20.3)	
Not at all	2 (3.1)	6 (9.4)	8 (6.3)	
**A6. I can allow health authority for inspection of mosquito larvae and fogging inside and outside the house**				5.3 (.06)
Extremely confident	53 (82.8)	61 (95.3)	114 (89.1)	
Moderately confident	9 (14.1)	2 (3.1)	11 (8.6)	
Not at all	2 (3.1)	1 (1.6)	3 (2.3)	
**A7. I can always convince my family to sleep under a mosquito net every day/night**				6.8 (.03)
Extremely confident	50 (78.1)	59 (92.2)	109 (85.2)	
Moderately confident	9 (14.1)	5 (7.8)	14 (10.9)	
Not at all	5 (7.8)	0 (0.0)	5 (3.9)	
**Summarized attitude level (good vs poor)**				.58 (.44)
Poor	18 (28.1)	22 (34.3)	40 (31.3)	
Good	46 (71.9)	42 (65.6)	88 (68.8)	

All *P*-values are based on a Chi-square test or Fisher's exact test (as appropriate) for independence analysis based on attitude characteristics regarding dengue (in respective study sites, separately). Attitude scores considered (≥80 = good and <80 = poor).

Additional file 6: Table S6. Summarized practice characteristics regarding dengue among study populations in selected urban and rural villages in northeastern Thailand (percentages in parentheses)

Factors	**Urban**	**Rural**	**Total**	x^2 (P $value$)

N (No of households)	**64**	**64**	**128**	
**Dengue prevention practices**				
**Bite prevention practices by the respondents**				
P1. Prevent mosquito-man contact	49 (76.6)	35 (54.7)	84 (65.6)	6.7 (.009)
P2. Use mosquito coils to reduce mosquitoes	31 (48.4)	34 (53.1)	65 (50.8)	.28 (.59)
P3. Use mosquito repellent/cream	29 (45.3)	28 (43.8)	57 (44.5)	.03 (.85)
P4. Use of smoke to drive away mosquitoes	16 (25.0)	20 (31.3)	36 (28.1)	.61 (.43)
P5. Covering body with clothe	30 (46.9)	46 (71.9)	76 (59.4)	8.2 (.004)
P6. Use window screens and bed net steps	46 (71.9)	32 (50.0)	78 (60.9)	6.4 (.01)
***Aedes* breeding sites**				
P7. What steps do you take to prevent mosquito breeding? during an outbreak?				
P8. Cleaning of garbage/trash	33 (51.6)	38 (59.4)	71 (55.5)	.79 (.37)
P9. Disposing water holding containers such as tires, parts of automobiles, plastic bottles, crack pots etc.	61 (95.3)	57 (89.1)	118 (92.2)	1.7 (.18)
P10. Cover tightly all water containers	41 (64.1)	43 (67.2)	84 (65.6)	.13 (.71)
P11. Frequently cleaning water filled containers and ditches around the house	36 (56.3)	43 (67.2)	79 (61.7)	1.6 (.20)
P12. Use insecticide sprays to reduce mosquitoes steps	39 (60.9)	26 (40.6)	65 (50.8)	5.2 (.02)
P13. Adding larvicide in water containers steps	48 (75.0)	52 (81.3)	100 (78.1)	.73 (.39)
P14. Remove water from flower pot trays steps	31 (48.4)	10 (15.6)	41 (32.0)	15.8 (.000)
P15. Change water in trays under the fridge steps	36 (56.3)	35 (54.7)	71 (55.5)	.03 (.85)
P16. Destroy / burn unused container side sprays steps	23 (35.9)	17 (26.6)	40 (31.3)	1.30 (.25)
**Summarized practice level (good vs poor)**				.64 (.42)
Poor	45 (70.3)	49 (76.6)	94 (73.4)	
Good	19 (29.7)	15 (23.4)	34 (26.6)	

All *P*-values are based on a Chi-square test or Fisher's exact test (as appropriate) for independence analysis based on practice characteristics regarding dengue (in respective study sites, separately). Practice scores considered (≥80 = good and <80 = poor).
